# Advancements and applications of gelatin-based scaffolds in dental engineering: a narrative review

**DOI:** 10.1007/s10266-025-01155-9

**Published:** 2025-07-17

**Authors:** Widya Lestari, Nining Irfanita, Muhammad Salahuddin Haris, Galvin Sim Siang Lin, Irwandi Jaswir, Deny Susanti Darnis, Nurul Ruziantee, Nurzafirah Mazlan, Erik Idrus, Lisa Rinanda Amir, Anis Fadhlina, Hassan I. Sheikh, Mohd Hafiz Arzmi

**Affiliations:** 1 Department of Fundamental Dental and Medical Sciences, Kulliyyah of Dentistry, International Islamic University Malaysia, Jalan Sultan Ahmad Shah, 25200 Kuantan, Pahang Malaysia; 2 International Institute for Halal Research and Training (INHART), International Islamic University Malaysia (IIUM), 53100 Jalan Gombak, Selangor Malaysia; 3 Department of Pharmacy, Faculty of Pharmacy and Health Sciences, Royal College of Medicine Perak, Universiti Kuala Lumpur, 30450 Ipoh, Perak Malaysia; 4 Department of Restorative Dentistry, Kulliyyah of Dentistry, International Islamic University Malaysia, Kuantan Campus, 25200 Kuantan, Pahang Malaysia; 5 Department of Chemistry, Kulliyyah of Science, International Islamic University Malaysia, Jalan Sultan Ahmad Shah, 25200 Kuantan, Pahang Malaysia; 6 Oceanography and Maritime Studies (INOCEM), Kulliyyah of Science, International Islamic University Malaysia, Bandar Indera Mahkota, 25200 Kuantan, Pahang Malaysia; 7 Department of Oral Maxillofacial Surgery & Oral Diagnosis, Kulliyyah of Dentistry, International Islamic University Malaysia, Bandar Indera Mahkota, 25200 Kuantan, Pahang Malaysia; 8 Borneo Marine Research Institute, Universiti Malaysia Sabah, 88400 Kota Kinabalu, Sabah Malaysia; 9 Department of Oral Biology, Faculty of Dentistry, Universitas Indonesia, Jawa Barat, 16424 Indonesia; 10 Faculty of Fisheries and Food Science, Universiti Malaysia Terengganu, 21030 Kuala Nerus, Terengganu Malaysia; 11 Cluster of Cancer Research IIUM (COCRII), International Islamic University Malaysia, 25200 Kuantan, Pahang Malaysia; 12 Melbourne Dental School, The University of Melbourne, Victoria, 3053 Australia

**Keywords:** Biomaterials, Endodontics, Gelatin, Periodontal regeneration, Scaffolds, Tissue engineering

## Abstract

Gelatin-based scaffolds have garnered significant attention in dental tissue engineering due to their biocompatibility, biodegradability, and resemblance to the extracellular matrix (ECM). This narrative review highlights recent advancements and applications of gelatin-based scaffolds for oral tissue regeneration. Various scaffold types, including hydrogels, electrospun nanofibers, hybrid composites, crosslinked matrices, and microspheres, are discussed in terms of their physicochemical characteristics, fabrication techniques, and regenerative potential. Gelatin methacryloyl (GelMA) hydrogels, for instance, exhibit favorable hydration and mechanical properties for endodontic regeneration, while electrospun nanofibers support enhanced cellular attachment and proliferation. Hybrid scaffolds incorporating ceramics, such as hydroxyapatite or β-tricalcium phosphate (β-TCP), improve mechanical strength, making them suitable for alveolar bone regeneration. Key parameters influencing scaffold performance, including gelatin concentration, crosslinking density, pore size, and biofunctionalization, are also examined. Applications span dentin–pulp complex regeneration, periodontal therapy, and bone defect repair. Despite their promise, limitations, such as rapid degradation and mechanical weakness, necessitate optimization either through chemical modification or composite formation. The integration of emerging technologies, including bioprinting and smart biomaterials, may further enhance scaffold functionality. This review underscores gelatin’s versatility and its pivotal role in shaping next-generation strategies for functional and biomimetic dental tissue restoration.

## Introduction

Oral tissue engineering is a multidisciplinary and dynamic field that encompasses the repair, restoration, and replacement of oral anatomy, such as teeth, gums, periodontal ligaments, alveolar bone, and salivary glands, which are impacted by oral health conditions and diseases. To overcome such concerns, various oral tissue engineering approaches have been employed, including biomaterial scaffolds, cell-based therapies, growth factors, and tissue engineering techniques. Among these, culturing stem cells onto scaffold surfaces has emerged as a cornerstone of oral tissue engineering in establishing a conducive platform for stem cell attachment, proliferation, and differentiation [1]. However, the presence of different tissues, such as tooth root, periodontium, dentin–pulp complex, blood vessels, and nerves, within the specific locale has made the tooth and the supporting tissue regeneration more arduous. The use of stem cells, growth factors, and biomaterials that mimic native cellular interactions and functional properties provides promising solutions to these challenges [2]. Various stem cell types obtained from specific tissues have been explored for dental tissue engineering. Dental-derived stem cells are especially beneficial due to their resemblance to mesenchymal stem cells (MSCs). Dental pulp stem cells (DPSCs), in particular, exhibit high proliferation, immunomodulatory capabilities, and the ability to regenerate dentin–pulp-like structures [3]. These cells express common MSC markers, such as CD29, CD44, CD73, CD90, CD105, CD106, CD117, CD146, and CD166, along with osteogenic markers like alkaline phosphatase (ALP), osteopontin (OPN), and osteocalcin (OCN) [3, 4]. Human dental pulp stem cells (hDPSCs) have shown the potential to differentiate into odontoblast-like cells involved in dentin formation, making them a promising candidate for pulp tissue regeneration [5]. Both in vitro and in vivo studies support the effectiveness of DPSCs in differentiating into mature odontoblasts and facilitating dental pulp regeneration when incorporated into scaffold systems [6, 7]. The first step in successful, biologically similar tooth regeneration is the selection of appropriate scaffold material, structure, and mechanical properties [8, 9]. An ideal scaffold should support cell growth, promote cell differentiation, and allow for the retention of cells at the target site. Cells are encapsulated using various polymers to protect them from immune response and loss during delivery to the target site while allowing for nutrients and waste to be diffused in and out of the particle [10]. A close match to the native extracellular matrix (ECM) of the target tissue is necessary for the scaffold to promote regeneration, differentiation, and function in the engineered tissue [11]. Natural and synthetic polymers have been explored in the fabrication of tissue engineering scaffolds. Natural polymers have the advantage of being compatible and functional with native tissue. Examples of such polymers include proteins (collagen and fibrin) and polysaccharides (chitosan, hyaluronic acid, alginate, etc.) [12–15]. Collagen type I is the dominant fibrous protein in hard tissues, including bone and dentin. This similarity to native tissue is advantageous in fabricating biomimetic scaffolds for tissue engineering [12].

Gelatin, a derivative of collagen, overcomes many of these limitations. It exhibits low antigenicity, while still preserving some informational cues from collagen that could potentially enhance cell attachment, differentiation, and proliferation, such as the Arg–Gly–Asp (RGD) sequence [17]. Gelatin is a heterogeneous mixture of peptides obtained from the partial degradation of collagen. Further enzymatic degradation of gelatin results in gelatin hydrolysate molecules [16]. The degradation of gelatin exposes active groups in the gelatin molecule and releases amino acids and small signaling molecules that promote cell proliferation [18]. Given the increasing focus on biologically inspired and functionally optimized scaffolds for dental pulp regeneration, a comprehensive synthesis of current evidence on gelatin-based scaffolds is warranted. While several polymers have been explored, gelatin offers a promising yet under-consolidated platform for dental tissue engineering. Hence, this narrative review aims to provide an overview of gelatin-based scaffolds employed in oral tissue engineering reported in recent literature, with a particular focus on advancements in gelatin-based scaffold development.

## Materials and methods

### Search strategy

A comprehensive digital literature search was conducted to identify relevant research articles published between January 2010 and April 2024. The electronic search was systematically performed across multiple scientific databases, including MEDLINE/PubMed, Web of Science, Scopus, and Google Scholar, to ensure a broad and inclusive retrieval of studies. The search strategy was limited to publications in the English language to maintain consistency in data interpretation and reporting. The primary search terms included combinations of keywords were also used where appropriate, depending on the database syntax and filters. Studies were screened for relevance based on titles and abstracts, followed by full-text assessment. Exclusion criteria included publications that did not present original research data, such as case reports, case series, editorials, commentaries, opinion pieces, and conference abstracts. Ultimately, 89 articles were included in this narrative review based on their relevance to the topic and contribution to the thematic analysis of gelatin-based biomaterials in dental tissue engineering.

### Properties and biomedical applications of gelatin in tissue engineering

Gelatin, a biodegradable and biocompatible polymer, is primarily composed of proteins (85–92%), mineral salts, and water [19]. Gelatin is the result of partial hydrolysis of collagen and is the predominant protein making up the natural ECM of various body tissues, such as skin, cartilage, tendons, and bones. Its biodegradability, biocompatibility, and cost-effectiveness have made it a popular choice for tissue engineering applications. Furthermore, gelatin does not elicit immunogenic reactions in the body, making it a superior material for biomedical applications [20]. Compared to collagen, gelatin contains fewer aromatic groups and exhibits improved solubility in aqueous solutions, allowing it to be incorporated with other natural and synthetic polymers, producing highly effective scaffolds [21]. In oral tissue engineering, hDPSCs have been combined with gelatin for oral tissue engineering applications, including dentin–pulp complex tissue engineering, dentin regeneration, and alveolar bone regeneration [2, 15, 22, 23]. Despite its many advantages, gelatin's physical crosslinking, mediated by hydrogen and peptide bonds, renders it thermos-reversible. In aqueous environments, gelatin absorbs large quantities of water at a rapid rate, causing it to lose structural integrity and degrade. Hence, the crosslinking of the gelatin polymer matrix is a crucial step for the successful implantation of the gelatin scaffold in vivo [24]. Crosslinking not only enhances gelatin's mechanical properties but also modifies its biochemical characteristics [25], and therefore, optimal crosslinking parameters need to be applied to prevent changes in gelatin bioactivity [24].

### Types of gelatin scaffolds

Gelatin scaffolds have garnered significant attention in tissue engineering and regenerative medicine due to their versatile applications, including wound healing [26], and the delivery of cells or bioactive substances [3]. Their fabrication process entails constructing three-dimensional structures from gelatin molecules and adjusting various factors for the final properties of the scaffold, including composition, structure, and function. Below, we explore some of the commonly reported types of gelatin scaffolds and their distinctive features:

### Gelatin hydrogels

Gelatin hydrogels (HG) are three-dimensional networks of gelatin molecules infused with water, making them highly suitable for hydration-dependent applications. The use of hydrogels in regenerative dentistry has been widely reported due to their ease of application and delivery into sites featuring irregular anatomy. Compared to other biomaterial variants, hydrogels present superior mechanical capabilities, porosity, enhanced biocompatibility, and customizable biodegradability [27]. HGs, whether simple or hybrid, have shown excellent chemical, mechanical, and biological activities [28, 29]. Furthermore, HGs have been reported to deliver cells to the target sites, where they degrade to allow new, healthy tissue to regenerate. Due to their porous nature, HGs can accommodate large amounts of water to facilitate the diffusion of cellular nutrients and waste. Additionally, HGs exhibit excellent elasticity and flexibility, mimicking the characteristics of the native ECM [30].

A notable variation of gelatin hydrogel is gelatin methacrylate (GelMA), which consists of additional methacryloyl (methacrylamide and methacrylate) side groups present in the gelatin molecule. GelMA has emerged as a recent innovation for replicating the 3D cellular microenvironment and fabricating 3D functional tissue constructs, owing to its biocompatibility, photo-crosslinkable properties, and moderate mechanical adjustability. It is noteworthy to highlight that GelMA hydrogel can be readily microfabricated using techniques, such as photopatterning, micro-molding, self-assembly, and bioprinting strategies. Khayat et al. [31] and Athirasala et al*.* [32] examined bulk GelMA hydrogels for dental pulp regeneration, and both studies showed that GelMA facilitated the attachment and proliferation of hDPSCs. Yang et al*.* [27] investigated the utilization of GelMA for producing hydrogel microspheres via the electrostatic microdroplet method and assessed the potential of GelMA microspheres in endodontic regeneration. The average size of GelMA microspheres encapsulating hDPSCs was approximately 200 µm, with a Young’s modulus of around 582.8 ± 66.0 Pa, closely resembling that of natural human dental pulp. The hDPSCs encapsulated within the microspheres were localized toward the outer layer of the microspheres and displayed efficient adherence, spreading, proliferation, and secretion of ECM proteins. Following subcutaneous implantation in a nude mouse model, the cell-laden GelMA microsphere group exhibited a higher formation of vascularized pulp-like tissues compared to the cell-laden bulk GelMA group, coupled with an appropriate degradation rate. The GelMA microspheres demonstrated impressive performance and significant potential as cell delivery carriers in endodontic regeneration [27].

### Electrospun gelatin nanofibers

Electrospinning is currently the predominant method for crafting three-dimensional porous structures with a substantial surface-to-volume ratio. Electrospun gelatin nanofibers are produced by electrospinning gelatin to create nano-fibrous scaffolds with extensive surface area and precise structural properties. The unique characteristic of electrospun gelatin nanofibers facilitates cell migration and substance diffusion (nutrients, water, and oxygen), thereby establishing a stable foundation for cell growth in tissue engineering applications [33]. Previously, Yang et al. [9] fabricated a zein/gelatin fiber electrospun gelatin nanofiber scaffold, assessed using human periodontal ligament stem cells. The addition of gelatin to zein improved the physicochemical properties of the scaffold, with increased hydrophilicity and cytocompatibility i.e., cell proliferation, affinity, and attachment recorded. The authors conclude that the hybrid gelatin–zein scaffold has good potential for use in tissue regeneration applications [9].

### Gelatin-based hybrid scaffolds

Hybrid gelatin scaffolds incorporate other substances into the gelatin scaffold, such as synthetic polymers or ceramics. The additional substance confers improved properties from the original substance to the scaffold [24]. Bone tissue is primarily collagen fibers and hydroxyapatite (HA) crystal complexes. Therefore, combining inorganic compounds into gelatin scaffolds for bone tissue regeneration significantly improves their performance. For example, the incorporation of HA and beta-tricalcium phosphate (TCP) particles into a gelatin scaffold produced a gelatin–HA–TCP scaffold with increased compressive strength, which is attributed to the increased density of the hybrid material from the addition of HA and TCP. In a study by Gu et al. [22], despite the increased density and strength of the hybrid biomaterial, scaffolds made with pure gelatin and gelatin–HA–TCP scaffolds shared a low elastic modulus and a remarkable elasticity. In bone defect filling applications, the gelatin–HA–TCP scaffold was amenable to deformation, allowing the material to more closely adhere to the irregular surfaces of the area with missing bone, resulting in an effective filling of the space [22].

### Crosslinked gelatin scaffolds

Crosslinking gelatin improves scaffold stability and mechanical integrity. It can be performed using either physical (ultraviolet irradiation, dehydrothermal treatment) or chemical (genipin, glutaraldehyde) means. During the crosslinking process, reaction sites are created via numerous free amino groups on the gelatin molecular chain. The sites undergo irreversible reactions, inducing gel–gel transition of gelatin, which improves the gelatin degradation rate in the human body. The application of crosslinking techniques ultimately aims to reduce the degradation of the gelatin scaffold in the body when used for biomedical applications [19, 24, 34–36].

### Gelatin microspheres and microparticles

Gelatin microspheres and microparticles carrying cells have interesting potential in endodontic regeneration applications due to their unique qualities [27, 37]. The small particle size of approximately 100–400 µm not only allows them to be easily delivered via injection into narrow and irregular root canals but also promotes the rapid exchange of nutrients and oxygen to maintain the viability of the cells post-implantation in the early ischemic environment [38]. Additionally, the microspheres enhanced cultured cells’ vitality and functionality to promote their development into microtissue over time within a biomimetic three-dimensional microenvironment [39]. A study’s findings also suggested that GelMA microspheres hold promising potential as cell carriers in endodontic regeneration [27].

### Factors affecting the properties of gelatin scaffolds

Numerous factors govern the efficacy of gelatin-based scaffolds in tissue engineering, such as gelatin concentration, crosslinking density, pore size and porosity, mechanical strength, biofunctionalization, and degradation rate. The gelatin concentration used during scaffold fabrication significantly influences its structural and functional characteristics. Specifically, higher gelatin concentrations tend to produce scaffolds with reduced pore size, enhanced mechanical integrity, and a slower rate of degradation [9, 32, 40]. Crosslinking, which involves the formation of irreversible bonds between free amino groups on gelatin chains, contributes to the stabilization and prolonged functionality of scaffolds within biological systems [22]. Common crosslinking agents include glutaraldehyde and genipin, which enhance scaffold durability [24, 34, 41]. A study by Nadeem et al. [24] demonstrated that scaffolds treated with dehydrothermal (DHT) processing followed by 24-h genipin immersion exhibited 20% less mass loss and a reduced degradation rate of 25% per week, in contrast to a 45% per week rate in DHT-only samples. This corresponds to a degradation timeline of 6–7 weeks for the genipin-treated scaffolds versus 2–3 weeks for the DHT-only group. Importantly, DHT treatment imparts sufficient structural strength to prevent scaffold collapse during genipin immersion, supporting a more stable and durable construct for tissue regeneration [24].

Pore dimension and arrangement are physical parameters that determine the porosity of the scaffold. Porosity affects the diffusion of nutrients from the scaffold, infiltration of cells, and ingrowth of tissue, ultimately affecting the migration and proliferation of adherent cells. According to a study, gelatin–HA–TCP scaffolds had pore sizes ranging from 20 to 200 µm, resulting in a highly porous and stable scaffold. At this pore size, the scaffold promotes cell adhesion and migration and provides pathways for the delivery of nutrients and metabolic waste to ensure tissue regeneration. The composite scaffolds prepared in this study had high water absorption properties and increased oxygen permeability due to the presence of hydrophilic groups, amino and carboxyl groups within the gelatin scaffold. The high water absorption points to optimal pore size within the gelatin scaffold [22]. Some mechanical properties associated with effective mechanical support in gelatin scaffolds for tissue regeneration include stiffness, elasticity, and strength. Gelatin concentration, crosslinking density, and scaffold architecture can be customized to produce scaffolds with the desired properties. Tensile strength refers to the material's resistance to fracture. In a study by Yang et al. [9], a gelatin–zein hybrid scaffold had better tensile resistance than zein alone albeit with a slight decrease in strain at break. Moreover, gelatin notably improves elasticity as in the case of nano-fibrous mats. This study indicates that at optimal concentrations, gelatin in zein improves stress resistance, but excess amounts could reduce strain, leading to brittleness [9].

Biofunctionalization refers to the process of introducing biological activity to a biomaterial. This process incorporates growth factors or biomimetic molecules into the biomaterial, which function as mediators in tissue engineering [42]. Some bioactive molecules used to biofunctionalize gelatin scaffolds to improve their bioactivity and confer specific cellular response functions to the scaffold include either growth factors, peptides, or ECM proteins. For example, an addition of 10 mg/mL fibronectin (FN) into a collagen/gelatin hydrogel formulation significantly improved the chemotactic and bioactive effects of the final biomaterial on human apical papilla cells (hAPCs). Higher concentrations of FN were associated with higher hAPC migration, viability, adhesion, spreading, and gene expression of pulp regeneration markers. The biofunctionalized scaffolds improved cell adhesion, proliferation, and differentiation for better tissue regeneration results [43]. Gelatin scaffolds degrade at different rates depending on the concentration of gelatin used, crosslinking density, or incorporation of other materials such as bioglass into the gelatin scaffold. The degradation rate is directly related to the duration of scaffold support and the timing of the tissue regeneration process. Optimizing for a slow and controlled degradation rate allows the scaffold to be stable and functional over longer durations. In a study on bioglass concentration and scaffold degradation, PBS solutions containing higher levels of bioglass showed a significantly decreased degradation rate [44]. Hydrogen bonds and ionic interactions between bioglass particles and water molecules reduced the interactions between gelatin macromolecule and water. Therefore, optimizing bioglass content may regulate the degradation rate and consequently, the strength of the scaffold in a physiological environment. It is crucial for scaffolds to degrade at a rate that aligns with the pace of tissue regeneration to ensure proper tissue integration and functional restoration [44].

### Gelatin scaffold applications in oral tissue engineering

Regeneration of the tooth and supporting tissue is a challenging process due to the complex tissue network involved, such as dentin–pulp complex, tooth root, periodontium, blood vessels, and nerves. Using biofunctional biomaterials containing pluripotent stem cells and growth factors that work synergistically to stimulate cellular activity, leading to tissue formation, is essential to the success of regeneration efforts [45]. Tissue regeneration is important within the dental and craniofacial realms for treating tissue traumas post-cancer surgery, skeletal disorders, congenital anomalies, and periodontal issues. Effective tissue regeneration ensures successful facial structure reconstruction necessary for proper cranio-dental function and harmonization with other sensory organs, such as eyes, nose, ears, and mouth. The use of specialized scaffolds in tissue engineering strategies allows cell adherence and growth in a three-dimensional microenvironment, ultimately leading to restored tissue functions [46].

Despite advances in biomaterial development for dental tissue regeneration scaffolds, single-component biomaterials perform poorly in supporting the attachment, proliferation, and differentiation of dental stem/progenitor cells. The introduction of novel biomimetic hybrid scaffolds aimed to improve the formation of dental tissue. Injectable gelatin hydrogels are an excellent example of an innovative biomaterial for use in dental tissue regeneration. The material has superb fluid properties, allowing its delivery via direct injection to the affected area, such as dental tissue, with minimal invasiveness [47, 48]. In another study, carboxyl-terminated poly(*N*-isopropylacrylamide) (PNIPAAm) was reacted with chitosan (CS), followed by varying gelatin (g) solution concentrations, crosslinked using genipin to produce CS-g-PNIPAAm [40]. The resulting PNIPAAm-g-CS copolymer/gelatin hybrid hydrogels were analyzed for chemical composition, microstructure, storage ability, loss modulus, and mechanical property using FT-IR, SEM, and rheological analysis, respectively. DPSCs were assessed for calcium deposition, ALP activity, toxicity, and osteogenic differentiation of hDPSCs cultured on the hydrogels using alizarin red staining, ALP test, live/dead assay, and real-time PCR, respectively. The analysis results showed that the hydrogel exhibited favorable characteristics, including porous structure, good flow rate, and enhanced cell viability. The properties supported effective osteogenic differentiation for dental tissue engineering [40].

### Gelatin-based scaffolds in endodontic regeneration

Several studies have reported the application of gelatin-based scaffolds for endodontic regeneration [19, 29, 31, 49, 50]. The application of hemostatic gelatin and fibrin-based hydrogel scaffolds for pulp regeneration in a minipig model was reported by Jang et al. [28]. The use of minipig models is widely documented in dental regenerative research to study pulp–dentin regeneration, vital pulp therapy, and periodontal bone tissue regeneration [28]. Immature premolars were extracted from minipigs as a model for regenerative endodontic therapy (RET), assessing radiographic and histologic outcomes over time. Treatment with gelatin-based hydrogel (GM) and positive control (PC) revealed the presence of pulp-like tissues and deposition of newly formed tertiary dentin with apex maturation in all experimental teeth. Furthermore, the GM group demonstrated favorable regenerative endodontic therapy (RET) outcomes in vivo, with no adverse changes such as inflammation or resorption observed in the immature tooth during the 12-week follow-up period. The findings from the study indicate that GM could serve as an appropriate scaffold material for promoting hemostasis in RET procedures [28]. Complementing these findings, Ducret et al. [89] reviewed fibrin-based scaffolds for dental pulp regeneration, noting their ability to support DP-MSC viability and promote collagen-rich pulp-like tissue formation in vitro and in vivo.

### Gelatin-based scaffolds in periodontal regeneration

Immense advancements in the field of periodontal regeneration research have been reported in the literature covering bone replacement grafts, soft tissue grafts, guided bone/tissue regeneration (GTR/GBR), root surface modification, delivery of growth factors, and gene therapy. Periodontal regeneration aims principally to restore degraded supporting tissues, such as alveolar bone, periodontal ligament, and cementum, surrounding a previously compromised tooth root. Developments in the use of gelatin for periodontal regeneration scaffolding are well-documented. Golubevas et al. [51] reported the use of dipentaerythritol hexa-acrylate (DPHA), ethylene glycol dimethacrylate (EGDMA), gelatin, and carbonated hydroxyapatite (cHAP) for the development of multi-composite scaffolds with different porosity by adjusting the polymer matrix-to-gelatin ratio at 50/50, 75/25, and 95/50, fabricated via copolymerization. The cHAP particles were embedded uniformly within the continuous polymeric matrix, with the scaffolds exhibiting voids and large agglomerate blocks in various regions of the composite pellets as shown via morphological analysis. Thermogravimetric analysis (TG) verified that the residual mass of inorganic material closely matched the starting amount of cHAP [51].

Computed tomography (CT) analysis revealed variations in average densities across the scaffolds, with higher densities observed in the central region compared to the outer layer. Hydrophilicity was found to be influenced by the composition and morphology, with the acrylate (75%)–gelatin (25%)–cHAP composite scaffold exhibiting the maximum wettability at 16.61. The favorable characteristics of the composites produced using this method hold promise for strategies involving porosity and hydrophilicity remodeling, ultimately facilitating scaffold-implant integration [51]. Therefore, it is proposed that these scaffolds hold promise for regenerating bone tissue in dental applications. Table 1 provides a summary of the progress made in developing scaffolds for dental and periodontal tissue regeneration, with a specific emphasis on those constructed from gelatin.

**Table 1:** Overview of Gelatin-Based Scaffolds in Dental Regeneration

Type of gelatin-based scaffold	Type of study	Effect/key findings	Application	Limitation	References
Alginate–Gelatin/nHA Microcapsules	In vitro	Increased ALP activity and calcium deposition in hDPSCs	Bone regeneration	–	Hasani-Sadrabadi et al. [2]
Electrospun Zein/Gelatin Scaffold	In vitro	Enhanced hydrophilicity, cell attachment, and cytocompatibility	Periodontal regeneration	Requires stable crosslinking	Yang et al. [9]
Alginate/Gelatin Hydrogel	In vitro	Enhanced proliferation and odontogenic differentiation of hDPSCs	Dental pulp regeneration	–	Yu et al. [15]
GelMA-based Photopolymerized Scaffold	In vitro	Supported viability of encapsulated cells; light exposure time critical	Pulp regeneration	Asymmetrical channel structure, curing issues	Monteiro et al. [19]
Electrospun GelMA + TCP Scaffold	In vitro / In vivo	Induced mineralization and pulp-like tissue in rat model	Vital pulp therapy	–	Han et al. [21]
Gelatin–hydroxyapatite tricalcium	In vitro	Promoted osteogenic differentiation and mineralization	Bone tissue engineering	–	Gu et al. [22]
CLN–nHA/CS–Gelatin Composite	In vitro	Supported cell adhesion and growth with porous structure	Bone tissue regeneration	–	Sadeghi et al. [23]
hDPSC-laden GelMA Microspheres	In vitro / In vivo	Promoted pulp-like vascularized tissue formation; high cell viability and ECM secretion	Endodontic regeneration	Reduced cell density in core region	Yang et al. [27]
GelMA-Encapsulated hDPSCs and HUVECs	In vitro / In vivo	Promoted pulp-like tissue with cell integration in dentin tubules	Endodontic regeneration	–	Khayat et al. [31]
Chitosan/Gelatin Scaffold	In vitro / In vivo	Induced osteogenic differentiation and mineralized matrix formation	Orofacial bone regeneration	rhBMP-2 dose not optimized	Bakopoulou et al. [34]
Injectable CS-g-PNIPAAm/Gelatin Hydrogel	In vitro	Enhanced ALP activity and osteogenic gene expression	Endodontic regeneration	–	Younesi et al. [40]
Collagen/Gelatin Hydrogel + Fibronectin	In vitro	Improved hAPC migration, adhesion, and pulp marker expression	Dental pulp regeneration	–	Matsumura et al. [43]
Fibroblast-encapsulated GelMA	In vitro	Higher fibroblast viability in collagen vs. GelMA; epithelial adhesion was not supported	Oral mucosa tissue engineering	Limited epithelial integration with GelMA	Tabatabaei et al. [80]
Gelatin-based Scaffold (Gelfoam)	In vivo	Supported mineralized tissue formation in immature necrotic teeth	Endodontic regeneration	No pulp/dentin formation observed	Londero et al. [81]
PLGA/Gelatin Electrospun Sheet (APES)	In vitro / In vivo	Guided pulp tissue regeneration in murine and porcine models	Endodontic regeneration	–	Chen et al. [85]
Gelatin–Hydroxyapatite Nanofibers	In vitro	Controlled degradation aligned with regeneration pace; bioactivity enhanced	Periodontal regeneration	–	Sharifi et al. [86]
3D Bioprinted GelMA–Calcium Silicate Scaffold	In vitro	Stimulated odontogenic differentiation of hDPSCs	Dentin/pulp tissue regeneration	Not tested in vivo	Yu et al. [88]

### Characteristics of various gelatin-based scaffolds

Table 2 provides a comparison of key characteristics and limitations among various gelatin-based scaffolds. Various types of gelatin-based scaffolds exhibit distinct structural forms and advantages tailored to specific dental applications. Gelatin hydrogels, known for their biocompatibility, injectability, and ECM-mimicking properties, are widely used in pulp regeneration but suffer from weak mechanical strength [31, 43]. Electrospun nanofibers provide a large surface-to-volume ratio and promote cell adhesion, making them suitable for periodontal repair, although they require crosslinking and have limited mechanical robustness [9]. Hybrid scaffolds, which combine gelatin with polymers or ceramics, offer enhanced mechanical strength and osteo-conductivity for bone and dentin regeneration but may pose fabrication challenges and immunogenic risks [21–23]. Crosslinked scaffolds improve structural stability and degradation control but can introduce cytotoxicity depending on the crosslinker [19]. Microspheres and microparticles are minimally invasive and effective for narrow anatomical sites, such as root canals, but may face challenges in cell loading [15, 27]. Furthermore, injectable hydrogels show promise in endodontic applications by supporting stem cell activity and mineralization although their mechanical performance remains a limitation without reinforcement [40].

**Table 2:** Comparative Properties of Different Types of Gelatin-Based Scaffolds

Scaffold type	Structural form	Key strengths	Common limitations	Common dental applications	References
Gelatin Hydrogels	Water-rich 3D matrix	High biocompatibility, injectability, flexibility and mimics ECM	Weak mechanical strength, rapid enzymatic degradation	Pulp regeneration, drug/cell delivery	Khayat et al. [31]; Leite et al. [43]; Tabatabaei et al. [80]
Electrospun Nanofibers	Nanofibrous mesh	Large surface-to-volume ratio Promotes cell adhesion and migration	Requires crosslinking, limited mechanical robustness	Periodontal tissue repair, wound healing	Yang et al. [9]; Chen et al. [85]
Hybrid Scaffolds	Composite with polymers or ceramics	Enhanced mechanical strength, osteo-conductivity, improved functionality	Complex fabrication, risk of immune reaction with some materials	Bone regeneration, dentin repair	Han et al. [21]; Gu et al. [22]; Sadeghinia et al. [23]; Bakopoulou et al. [34]
Crosslinked Scaffolds	Stabilized network (chemical/physical)	Improved stability, slower degradation, tailored properties	Potential cytotoxicity (e.g., with glutaraldehyde)	General dental scaffold applications	Monteiro et al. [19]; Sharifi et al. [86]
Microspheres/Microparticles	Injectable spherical carriers	Minimally invasive, uniform cell/drug distribution, suitable for confined spaces	Low cell loading, fabrication complexity	Endodontic regeneration, narrow root canal applications	Yang et al. [27]; Yu et al. [15]; Choi et al. [88]
Injectable Hydrogels	Thermosensitive injectable gel	Supports hDPSC proliferation and osteogenic gene expression	Limited mechanical integrity without secondary reinforcement	Endodontic regeneration	Samiei et al. [40]
Sponge-like Scaffold	Absorbent matrix	Promotes mineralized tissue formation in immature teeth (in vivo)	Limited pulp-dentin regeneration	Regenerative endodontics	Londero et al. [81]

## Discussion

Gelatin-based scaffolds have emerged as transformative biomaterials in oral tissue engineering, showcasing their versatility and effectiveness in addressing the diverse challenges associated with regenerating oral and dental tissues. A comprehensive understanding of gelatin, its properties, and its specific requirements is essential for developing gelatin-based materials tailored to targeted applications. Owing to its chemical resemblance to the ECM of connective tissues, gelatin has been extensively studied as a biopolymer in regenerative medicine and dentistry. Gelatin is derived through the denaturation and hydrolysis of collagen, a primary structural component of the ECM. The ECM itself is an acellular three-dimensional network composed of diverse amino acid- and sugar-based macromolecules. It plays a critical role in cellular and tissue dynamics by providing structural support, facilitating cell adhesion, and regulating cell function and morphogenesis. In addition, the ECM contributes to tissue organization and homeostasis by enabling the diffusion of nutrients, metabolites, and growth factors [52].

Gelatin is an exceptional biomaterial due to its unique properties, including biodegradability, biocompatibility, the ability to form hydrogels, and the presence of accessible functional groups that facilitate chemical modifications with other biomaterials or biomolecules. Gelatin comprises fewer aromatic groups and shows better quality in terms of solubility in aqueous solutions, permitting it to be combined with other natural and synthetic polymers, producing highly effective scaffolds as compared to collagen [21]. In oral tissue engineering, hDPSCs have been incorporated with gelatin for oral tissue engineering applications, including dentin–pulp complex tissue engineering, dentin regeneration, and alveolar bone regeneration [2, 15, 22, 23]. In aqueous environments, gelatin can absorb a large amount of water rapidly, which leads to loss of structural integrity and degradation. Hence, the successful implantation of the gelatin scaffolds in vivo can be achieved with the presence of crosslinked gelatin polymer matrix [24]. Furthermore, gelatin is highly suitable for creating three-dimensional (3D) cell culture models. Saotome et al*.* [53] evaluated the feasibility of gelatin hydrogel nonwoven fabrics (GHNF) as a 3D scaffold and reported promising results. GHNF demonstrated the capacity to support homogeneous cell proliferation within the scaffold, efficient ECM secretion, and enhanced cell survival by reducing apoptosis. When GHNF is transplanted into tissue defects, its interconnected porous structure facilitates the infiltration of stromal cells and endothelial progenitor cells, thereby promoting angiogenesis. Consequently, ECM production and the subsequent tissue formation are also expected to be promoted [53]. A key advantage of gelatin is the presence of arginine–glycine–aspartic (RGD) motifs in its polymer structure. These motifs play an important role in promoting cell adhesion, distinguishing gelatin from other polymers in terms of its biological properties [54]. RGD sequences, commonly found on many ECM and plasma proteins, are essential for cell recognition and adhesion. Their importance extends beyond tissue engineering to applications in tumor therapy, where they are utilized through recombinant techniques and chemical methods [55]. Moreover, gelatin has a flexible amino acidic structure that presents several free functional groups, enabling chemical conjugation and structural modifications. This adaptability allows researchers to tailor gelatin-based materials for specific biomedical applications, making it a versatile platform in tissue engineering [56].

ECMs consist of an acellular 3D network of various amino acid- and sugar‐based macromolecules. These matrices bring cells together, support them, control tissue structures, regulate cell function and morphogenesis, and facilitate the diffusion of nutrients, metabolites, and growth factors [52]. Among biomaterials, gelatin stands out as one of the most commonly used in 3D cell culture due to its ability to provide both chemical and biological cues, creating a conducive environment for hosting various cell types [57]. Despite its advantages, gelatin exhibits several limitations, including poor mechanical strength, rapid enzymatic degradation, and low solubility in concentrated aqueous solutions, which restrict its broader application [58]. To improve the mechanical strength of gelatin, polysaccharides are often added to formulations. The resulting gelatin–polysaccharide matrix not only mimics glycoproteins found in the ECM but also introduces synergistic properties that neither material possesses individually [36, 59, 60]. This strategy may serve as a powerful tool for designing complex hybrid polymeric frameworks in a broad spectrum of tissue engineering applications. For instance, Bakopoulou et al*.* [34] combined gelatin and chitosan to enhance orofacial bone regeneration. Both scaffold types supported cell viability and proliferation, accompanied by extensive formation of a hydroxyapatite-rich nano-crystalline calcium phosphate phase. Interestingly, differential gene expression analysis revealed that the concentration of glutaraldehyde used during crosslinking significantly influenced the expression of osteo-/odontogenic genes [34]. Thus, these findings highlight the importance of fine-tuning crosslinking parameters to optimize scaffold performance for specific regenerative applications. Mechanical properties of gelatin can be enhanced, and biochemical properties can be modified by means of crosslinking [25]. Hence, applied crosslinking parameters should be optimal in preventing changes of gelatin bioactivity [24].

Gelatin–polysaccharide hydrogels can absorb large amounts of water, typically more than 100 times their dry mass. This enables them to function as matrix-inspired platforms in tissue engineering studies for mammalian cell adhesion and growth, infiltration, and tissue vascularization [61]. In hybrid hydrogel formulations, polysaccharides provide stability to the scaffolds, while the gelatin component enhances biological performance. A variety of molecular weights, structures (e.g., linear or branched), functionality (monofunctional or polyfunctional), and water affinity and solubility of polysaccharides used in hybrid hydrogel formulations allows the library of ECM‐mimicking biomaterials to be expanded. The addition of protein components induces gel formation through a broad range of chemical and physical interactions, including electrostatic, hydrophobic, and hydrogen bonding. Gelatin–polysaccharide hydrogels are biodegradable, biocompatible, and generally recognized as safe (GRAS), facilitating their transition from research to clinical applications. The formulations can be prepared using various approaches, such as electrospinning [21], film casting [12], physical mixing [62], layer‐by‐layer assembly [56], and freeze-drying [63].

Traditionally, two-dimensional (2D) cell culture systems have been the primary approach for studying cell behavior and function in tissue engineering. Nevertheless, the advent of highly stable, functional 3D cell culture systems has improved observations of cell–cell and cell–ECM interactions [64]. Hydrogel formulations optimized for physical, biochemical, and mechanical properties can recreate biomimetic 3D ECM scaffolds that more closely resemble the in vivo cell environment [29]. This capability enables studies, such as drug efficacy testing, to be conducted in highly biomimetic environments, yielding more accurate and clinically relevant results [65]. The selection of the polymer(s) and cross-linking method(s) in hydrogel preparation is a crucial factor to produce a 3D microenvironment scaffold with the desired properties and guide a desired cellular response to achieve a 3D biomimetic construct [66]. HGs, whether simple or hybrid, have shown outstanding chemical, mechanical, and biological activities [28, 29]. Furthermore, HGs have been reported in delivery cells to target sites, allowing degradation in regenerating new and healthy cells. Due to their porous nature, HGs possess the ability to accommodate a large amount of water in facilitating the diffusion of cellular nutrients and waste. Furthermore, HGs have displayed remarkable in terms of elasticity and flexibility, mirroring the attributes of the native ECM [30].

A distinct variation of gelatin hydrogel is GelMA, which comprises additional methacryloyl (methacrylamide and methacrylate) side groups exhibited in the gelatin molecule. GelMA has appeared as a cutting-edge solution for imitating the 3D cellular microenvironment and developing 3D functional tissue constructs as a consequence of its biocompatibility, photo-crosslinkable properties, and moderate mechanical flexibility. Techniques, such as photopatterning, micro-molding, self-assembly, and bioprinting strategies, can be applied to fabricate GelMA hydrogel. Khayat et al. [31] and Athirasala et al. [32] discovered the successful application of GelMA hydrogels for dental pulp regeneration as it expedited the attachment and proliferation of hDPSCs. Meanwhile, Yang et al. [27] identified the potential of GelMA microspheres in the regeneration of the pulp–dentin complex via the electrostatic microdroplet method.

Electrospinning is one of the most versatile and promising techniques for producing nano-fibrous mats with desirable properties, including a large surface-to-volume ratio, high mechanical strength, flexibility, small pore size, and ease of functionalization [21, 67]. Nanofibers produced using this method have a wide range of applications in tissue engineering, wound dressing, vascular grafting, and drug delivery systems [21, 68]. An important factor in the electrospinning process is the choice of solvent, which affects viscosity, electrostatic potential, and temperature. Organic solvent systems are commonly employed for electrospinning biopolymers, such as gelatin, collagen, chitosan, chitin, and various synthetic polymers, enabling the production of high-quality nanofibers [69].

Electrospun gelatin nanofibers have a unique characteristic in facilitating the migration of cells and diffusion of substances (nutrients, water, and oxygen), thus laying a firm foundation for cellular growth in tissue engineering applications [33]. Previously, Yang et al. [9] have engineered zein/gelatin fiber electrospun gelatin nanofiber scaffold, evaluated by means of human periodontal ligament stem cells. As a result, the physicochemical properties of the scaffold, with increased hydrophilicity and cytocompatibility, i.e., cell proliferation, affinity, and attachment, have improved. Therefore, it is worth mentioning that the hybrid gelatin–zein scaffold exhibited great potential to generate tissue [9].

Crosslinking methods can be categorized into physical, biological, and chemical methods. Among chemical methods, commonly used chemical crosslinking agents include the aldehydes formaldehyde, glutaraldehyde, glyceraldehyde, glyoxal, polyepoxy compounds, and carbodiimides [34, 36], while natural crosslinking agents, like genipin, are also employed due to their lower toxicity profile [36]. Stability of mechanical integrity of a scaffold can be improved by the crosslinking of gelatin, which can be performed via physical (ultraviolet irradiation, dehydrothermal treatment) or chemical (genipin, glutaraldehyde) means. Reaction sites are created through numerous free amino groups on the molecular chain of gelatin during the crosslinking procedure, which leads to improved gelatin degradation rate in the human body. The ultimate goal is to reduce the degradation of gelatin scaffold in the body pertaining to biomedical applications [19, 24, 34–36]. In tissue regeneration, bone substitutes benefit from improved mechanical properties with chemical crosslinking; however, the use of chemicals often triggers toxic and adverse reactions. The use of synthetic polymers offers more controlled synthesis although the byproducts generated in the process can be cytotoxic [70]. An alternative to chemical crosslinking gelatin is physical crosslinking, often using UV light or microwave energy to rearrange the amino acid sequences in gelatin [19, 58]. Despite their simplicity, these techniques are less desirable due to the difficulty in achieving precise control over crosslinking density, which can affect the scaffold's mechanical and biological properties. The challenges associated with crosslinking gelatin have limited its use in in situ hydrogel formulations. However, crosslinking improves the mechanical properties of gelatin in wound dressing formulations, reducing its solubility and degradation in an aqueous environment. The degree and nature of crosslinking (e.g., amenability of the crosslink bond to hydrolytic degradation) allow gelatin to adopt various physical forms with differing mechanical and rheological properties, ranging from amorphous gels to semi-stiff sheets. Consequently, selecting appropriate crosslinking agents and protocols is critical for tailoring gelatin’s properties, particularly in the production of both layers of a bilayer dressing [71].

GelMA is a well-established biomaterial produced by modifying the amine-containing side groups of naturally derived gelatin (denatured collagen) with a methacrylate group. Typically, the GelMA is cured with light and can be tailored to exhibit various physical and mechanical characteristics [19]. The functionalization of gelatin to produce GelMA was first described by Van Den Bulcke et al*.* [72] using unsaturated methacrylamide groups. The final semi-synthetic hydrogel has superior mechanical properties compared to gelatin while maintaining its biological characteristics. This has facilitated its use in various tissue engineering applications, including in bones, myocardium, cardiac tissues, cartilage, vascular networks, skeletal muscle, and more, where it supports cellular interactions and degrades upon exposure to matrix metalloproteinases. One limitation of pure GelMA is its poor mineralization potential, limiting its use in pure form for pulp capping. To address this, the biologically active ceramic additives bioglass and calcium phosphates (e.g., TCP) are incorporated into GelMA formulations [21]. Beta-tricalcium phosphate [Ca_3_(PO_4_)^2^], an osteoconductive, osteo-inductive, and cell-mediated resorbed synthetic bone graft substitute, is a commonly used additive with GelMA that induces a remarkable tissue response [21]. Efforts to optimize the mechanical and biocompatible features of GelMA formulations for dental tissue applications are ongoing, using various combinations of polymers and ceramics.

Researchers have shown high interest in developing hydrogels as important biomaterials for pulp regeneration, primarily due to their ease of application at the target site and conformity to sites with irregular anatomy [31, 73]. Hydrogels are also highly humid and possess highly plastic properties, conferring them very similar structural properties to the ECM [58]. Within tissue engineering strategies, collagen and gelatin have been used successfully to mimic connective tissue [32, 74]. Biocompatible hydrogels inserted into damaged tissues degrade gradually, allowing for their replacement by newly regenerated tissue. This characteristic, coupled with their ability to incorporate specific signaling agents, highlights their potential for guided pulp regeneration [43].

Pore microstructure is a key factor determining scaffold efficacy; this includes pore size, porosity, interconnection between pores, and surface/volume ratio. Efficient scaffolds are highly porous, have suitable pore sizes to support cell migration, cell proliferation, and vascularization deep inside the pores, and are permeable to facilitate blood vessel ingrowth, transportation of nutrients, and removal of waste products [75].

Owing to unique qualities, gelatin microspheres and microparticles carrying cells exhibit significant potential in endodontic regeneration [27, 37]. The small particles, approximately 100–400 µm in size, lead to improved delivery of cells via injection into narrow and irregular root canals as well as promoting the rapid exchange of nutrients and oxygen to maintain cell viability in the early ischemic environment [38]. Hence, cultured cells' vitality and functionality improved [39]. GelMA microspheres have been shown to possess promising potential as cell carriers in endodontic regeneration, as found by a study [27]. During cell culture, scaffold swelling enhances cell infiltration in three dimensions by increasing pore size and overall porosity, thereby maximizing the scaffold's internal surface area. Higher cell infiltration has been recorded in scaffolds with higher degrees of swelling due to the larger surface area to volume ratio. The addition of nHA and PANI affected the swelling behavior of scaffolds, where a barrier layer is formed on the surface, preventing water entry into the scaffold. The interaction between polymer chains nHA and PANI uses hydrophilic groups of macromolecules to decrease solvent uptake, resulting in reduced swelling. The swelling behavior has an important effect on the mechanical properties of the final scaffold [41]. Therefore, careful consideration of scaffold components during formulation is crucial as they directly influence water uptake and swelling behavior, which in turn affect the scaffold's structural integrity and biological performance.

The density of a scaffold is directly related to its porosity and affects its mechanical strength, permeability, and presence of structural defects [76]. As mentioned previously, porosity is a key consideration in scaffold formulation, as it directly controls the mass transfer of nutrients and metabolic waste products to cells, and assists cell migration and vascularization [75]. Together, these factors determine the success of new tissue formation. Increased scaffold density results in a product with increased mechanical strength. Meanwhile, a high porosity provides a favorable biological environment for cell culture; hence, a balance between the porosity and density of a scaffold must be decided according to specific requirements and applications [77].

Another crucial characteristic of scaffolds used in tissue regeneration applications is their swelling behavior. The swelling behavior directly affects the ability of the scaffold to absorb body fluids, transport important nutrients and metabolites, and exert mechanical stress on surrounding tissues [40]. The GelMA scaffolds demonstrated a progressive swelling increase in the first hour, reaching a steady state later. This swelling behavior is attributed to the hydrophilic nature of the GelMA material, which is enhanced by the hydrophilic TCP ceramic particles in the formulation. Although the developed scaffolds had similar degradation patterns, the TCP-laden samples degraded at a much faster rate. This pattern of degradation pattern is desirable as it facilitates the gradual release of essential minerals, such as Ca^2+^ and PO_4_^3−^, into the surrounding environment. These minerals provide the necessary elements for successful bone remodeling in bone regeneration applications [21].

Studies on gelatin-based scaffolds reveal varying outcomes depending on scaffold composition and fabrication. Yang et al. [9] demonstrated enhanced cell attachment using electrospun zein/gelatin nanofibers, while Gu et al. [22] showed improved mechanical strength in gelatin–HA–TCP hybrids for bone regeneration. Similarly, GelMA microspheres studied by Yang et al. [27] promoted superior vascularized pulp-like tissue formation compared to bulk GelMA, likely due to better nutrient diffusion and surface area. These comparisons emphasize that scaffold type, structure, and additive materials critically influence regenerative performance and should be tailored to specific dental applications.

Despite the remarkable progress, several challenges remain in the clinical translation of gelatin-based scaffolds. One major limitation is the thermos-reversibility of gelatin, which can lead to structural instability in aqueous environments. While crosslinking improves stability, it may also reduce the bioactivity of the scaffold, necessitating a careful balance between mechanical and biological properties. Another challenge lies in the scalability and reproducibility of scaffold fabrication techniques. Methods, such as electrospinning and bioprinting, require precision and standardization to ensure consistent scaffold quality. In addition, the cost of high-purity gelatin and crosslinking agents may limit their widespread application in resource-constrained settings. The scientific contribution of gelatin-based scaffolds is primarily demonstrated in Table 2. This table collectively demonstrate the broad applicability of gelatin-based scaffolds in dental regenerative strategies, each tailored to specific tissue targets. Gelatin methacryloyl (GelMA) emerged as a prominent material due to its cytocompatibility and ease of photopolymerization, supporting fibroblast viability [80], dental pulp stem cell encapsulation [31], and in situ polymerization under clinical conditions [19]. Innovative designs, such as microsphere platforms [27] and bioprinted hydrogel composites [15], further optimized cell delivery and bioactivity. For periodontal applications, gelatin blended with zein enhanced cellular attachment [9], while incorporation of hydroxyapatite and tricalcium phosphate improved osteogenic differentiation and scaffold stability [22, 86]. Additionally, chitosan gelatin constructs showed promise in orofacial bone repair by fostering favorable stem cell responses [34]. Despite differing in scaffold architecture and cellular targets, all studies underscored the biological compatibility and regenerative support provided by gelatin-based matrices. These findings affirm gelatin’s value as a customizable and effective scaffold material for advancing dental tissue engineering. These comparative insights reinforce the need for application-specific scaffold customization and suggest that no single gelatin-based system is universally superior. Rather, tailored combinations or hybrid approaches may be required to optimize both biological compatibility and mechanical performance across different dental tissue engineering scenarios.

Future research should focus on addressing the limitations of gelatin-based scaffolds while exploring innovative applications in oral tissue engineering. The integration of emerging technologies, such as 4D bioprinting and microfluidics, could enable the production of more complex and functional scaffolds. These techniques allow for precise control over scaffold architecture and the incorporation of multiple cell types or bioactive molecules [78]. Moreover, researchers could explore the development of smart scaffolds that respond to environmental stimuli, such as pH or temperature, which could enhance their functionality. For instance, stimuli-responsive scaffolds could release growth factors or drugs in a controlled manner, improving therapeutic outcomes. Future research should also look into tailoring scaffold properties to individual patient needs through the use of patient-derived cells and biomimetic materials, which could improve the efficacy and safety of tissue regeneration therapies [79].

The advancements in gelatin-based scaffolds have far-reaching implications for oral tissue engineering. By providing a versatile and biocompatible platform for tissue regeneration, these scaffolds have the potential to revolutionize the treatment of dental and craniofacial defects. Their ability to mimic the native ECM and support cellular activity aligns with the principles of regenerative medicine, offering a more natural and sustainable approach to tissue repair. While in vitro and animal studies have demonstrated the potential of gelatin-based scaffolds, more extensive preclinical and clinical studies are needed to validate their safety and efficacy in humans. As research progresses, these scaffolds could play a central role in addressing the unmet needs of patients with complex dental and craniofacial conditions.

## Conclusion

Tissue engineering offers innovative solutions for dental tissue repair and regeneration. Gelatin-based scaffolds show great promise due to their biocompatibility, biodegradability, and structural similarity to the ECM, supporting cell adhesion, growth, and differentiation. However, despite these advantages, gelatin-based scaffolds also present several limitations that must be considered in clinical contexts. These include rapid biodegradability, relatively poor mechanical strength, and challenges in achieving consistent scaffold fabrication. Owing to the complexity of tissue networks, such as dentin–pulp complex, tooth root, periodontium, blood vessels, and nerves, the regeneration of the tooth and tissue is challenging. Gelatin-based scaffold can be a promising biomaterial since it is considered biofunctional, which can be well integrated with living tissues without causing toxicity. In fact, gelatin can be loaded with pluripotent stem cells and growth factors that work synergistically in stimulating cellular activity, leading to the formation of tissue, which is crucial to the regeneration efforts. Through ongoing progress in biomaterials science, cell biology, and regenerative medicine, approaches based on gelatin scaffolds embrace the dental care revolution as they provide more efficient and patient-centered solutions. To fully harness their clinical utility, future efforts must also address these practical barriers to ensure safety, scalability, and long-term functionality. Hence, diverse dental problems can be managed, and the oral health and well-being of patients can be rejuvenated.

## Data Availability

Raw data supporting the findings of this study are available from the corresponding author on request.

## References

[1] Vasanthan KS, Sybil D, Shrivastava PK, Ahmad T. Utilization of gelatin methacryloyl scaffolds in the regeneration of tooth and its supporting tissues. Int J Sci Reports. 2022;8:260.

[2] Alipour M, Firouzi N, Aghazadeh Z, Samiei M, Montazersaheb S, Khoshfetrat AB, et al. The osteogenic differentiation of human dental pulp stem cells in alginate-gelatin/Nano-hydroxyapatite microcapsules. BMC Biotechnol. 2021;21:1–12. 33430842 10.1186/s12896-020-00666-3 PMC7802203

[3] Moonesi Rad R, Atila D, Akgün EE, Evis Z, Keskin D, Tezcaner A. Evaluation of human dental pulp stem cells behavior on a novel nanobiocomposite scaffold prepared for regenerative endodontics. Mater Sci Eng C. 2019;100:928–48. 10.1016/j.msec.2019.03.022 30948129

[4] Khazaei M, Bozorgi A, Khazaei S, Khademi A. Stem cells in dentistry, sources, and applications. Dent Hypotheses. 2016;7:42–52.

[5] Tatullo M, Ambrogio G, Sammartino G. Advances in dental implants, tissue engineering and prosthetic materials. Mater (Basel). 2023;16:5871. 10.3390/ma16175871 PMC10489074 37687564

[6] Atalayin C, Tezel H, Dagci T, Yavasoglu NUK, Oktem G, Kose T. In vivo performance of different scaffolds for dental pulp stem cells induced for odontogenic differentiation. Braz Oral Res. 2016;30:1–7. 10.1590/1807-3107BOR-2016.vol30.0120 27901202

[7] Chamieh F, Collignon AM, Coyac BR, Lesieur J, Ribes S, Sadoine J, et al. Accelerated craniofacial bone regeneration through dense collagen gel scaffolds seeded with dental pulp stem cells. Sci Rep. 2016;6:1–11. 27934940 10.1038/srep38814 PMC5146967

[8] Lawrence BJ, Madihally SV. Cell colonization in degradable 3D porous matrices. Cell Adh Migr. 2008;2:9–16. 19262124 10.4161/cam.2.1.5884 PMC2634998

[9] Yang F, Miao Y, Wang Y, Zhang LM, Lin X. Electrospun zein/gelatin scaffold-enhanced cell attachment and growth of human periodontal ligament stem cells. Materials (Basel). 2017;10:1–14. 10.3390/ma10101168 PMC5666974 29023390

[10] Huang X, Zhang X, Wang X, Wang C, Tang B. Microenvironment of alginate-based microcapsules for cell culture and tissue engineering. J Biosci Bioeng. 2012;114:1–8. 22561878 10.1016/j.jbiosc.2012.02.024

[11] Khan F, Dahman Y. A novel approach for the utilization of biocellulose nanofibres in polyurethane nanocomposites for potential applications in bone tissue implants. Des Monomers Polym. 2012;15:1–29.

[12] Erturk PA, Altuntas S, Irmak G, Buyukserin F. Bioinspired collagen/gelatin nanopillared films as a potential implant coating material. ACS Appl Bio Mater. 2022;5:4913–21. 36203409 10.1021/acsabm.2c00633 PMC9580019

[13] Ranjbar J, Koosha M, Chi H, Ghasemi A, Zare F, Abdollahifar MA, et al. Novel chitosan/gelatin/oxidized cellulose sponges as absorbable hemostatic agents. Cellulose. 2021;28:3663–75.

[14] Xiao S, Zhao T, Wang J, Wang C, Du J, Ying L, et al. Gelatin methacrylate (GelMA)-based hydrogels for cell transplantation: an effective strategy for tissue engineering. Stem Cell Rev Reports. 2019;15:664–79. 10.1007/s12015-019-09893-4 31154619

[15] Yu H, Zhang X, Song W, Pan T, Wang H, Ning T, et al. Effects of 3-dimensional bioprinting alginate/gelatin hydrogel scaffold extract on proliferation and differentiation of human dental pulp stem cells. J Endod. 2019;45:706–15. 31056297 10.1016/j.joen.2019.03.004

[16] Liu D, Nikoo M, Boran G, Zhou P, Regenstein JM. Collagen and gelatin. Annu Rev Food Sci Technol. 2015;6:527–57. 25884286 10.1146/annurev-food-031414-111800

[17] Yamada KM. Adhesive recognition sequences. J Biol Chem. 1991;266:12809–12. 2071570

[18] Chandel NS. Amino acid metabolism. Cold Spring Harb Perspect Biol. 2021;13:1–17. 10.1101/cshperspect.a040584 PMC8015690 33795250

[19] Monteiro N, Thrivikraman G, Athirasala A, Tahayeri A, França CM, Ferracane JL, et al. Photopolymerization of cell-laden gelatin methacryloyl hydrogels using a dental curing light for regenerative dentistry. Dent Mater. 2018;34:389–99. 29199008 10.1016/j.dental.2017.11.020 PMC5818302

[20] Ramshaw JAM, Werkmeister JA, Glattauer V. Collagen-based biomaterials. Biotechnol Genet Eng Rev. 1996;13:335–82. 8948117 10.1080/02648725.1996.10647934

[21] Han Y, Dal-Fabbro R, Mahmoud AH, Rahimnejad M, Xu J, Castilho M, et al. GelMA/TCP nanocomposite scaffold for vital pulp therapy. Acta Biomater. 2024;173:495–508. 37939819 10.1016/j.actbio.2023.11.005 PMC10964899

[22] Gu Y, Bai Y, Zhang D. Osteogenic stimulation of human dental pulp stem cells with a novel gelatin-hydroxyapatite-tricalcium phosphate scaffold. J Biomed Mater Res - Part A. 2018;106:1851–61. 10.1002/jbm.a.36388 29520937

[23] Sadeghinia A, Soltani S, Aghazadeh M, Khalilifard J, Davaran S. Design and fabrication of clinoptilolite–nanohydroxyapatite/chitosan–gelatin composite scaffold and evaluation of its effects on bone tissue engineering. J Biomed Mater Res—Part A. 2020;108:221–33. 10.1002/jbm.a.36806 31581359

[24] Nadeem D, Kiamehr M, Yang X, Su B. Fabrication and in vitro evaluation of a sponge-like bioactive-glass/gelatin composite scaffold for bone tissue engineering. Mater Sci Eng C. 2013;33:2669–78. 10.1016/j.msec.2013.02.021 23623083

[25] Bello AB, Kim D, Kim D, Park H, Lee SH. Engineering and functionalization of gelatin biomaterials: from cell culture to medical applications. Tissue Eng—Part B Rev. 2020;26:164–80. 31910095 10.1089/ten.TEB.2019.0256

[26] Kang BS, Na YC, Jin YW. Comparison of the wound healing effect of cellulose and gelatin: an in vivo study. Arch Plast Surg. 2012;39:317–21. 22872833 10.5999/aps.2012.39.4.317 PMC3408275

[27] Yang T, Zhang Q, Xie L, Zhang R, Qian R, Tian Y, et al. hDPSC-laden GelMA microspheres fabricated using electrostatic microdroplet method for endodontic regeneration. Mater Sci Eng C. 2021;121: 111850. 10.1016/j.msec.2020.111850 33579484

[28] Jang JH, Moon JH, Kim SG, Kim SY. Pulp regeneration with hemostatic matrices as a scaffold in an immature tooth minipig model. Sci Rep. 2020;10:1–11. 32719323 10.1038/s41598-020-69437-6 PMC7385085

[29] Kang HJ, Park SS, Saleh T, Ahn KM, Lee BT. In vitro and in vivo evaluation of Ca/P-hyaluronic acid/gelatin based novel dental plugs for one-step socket preservation. Mater Des. 2020;194: 108891.

[30] Mantha S, Pillai S, Khayambashi P, Upadhyay A, Zhang Y. Smart hydrogels in tissue engineering and regenerative medicine. Materials (Basel). 2019;12:33. 10.3390/ma12203323 PMC6829293 31614735

[31] Khayat A, Monteiro N, Smith EE, Pagni S, Zhang W, Khademhosseini A, et al. GelMA-encapsulated hDPSCs and HUVECs for dental pulp regeneration. J Dent Res. 2017;96:192–9. 28106508 10.1177/0022034516682005 PMC5331619

[32] Athirasala A, Lins F, Tahayeri A, Hinds M, Smith AJ, Sedgley C, et al. A Novel strategy to engineer pre-vascularized full-length dental pulp-like tissue constructs. Sci Rep. 2017;7:1–11. 28607361 10.1038/s41598-017-02532-3 PMC5468292

[33] Kucinska-Lipka J, Gubanska I, Janik H, Sienkiewicz M. Fabrication of polyurethane and polyurethane based composite fibres by the electrospinning technique for soft tissue engineering of cardiovascular system. Mater Sci Eng C. 2015;46:166–76. 10.1016/j.msec.2014.10.027 25491973

[34] Bakopoulou A, Georgopoulou Α, Grivas I, Bekiari C, Prymak O, Loza Κ, et al. Dental pulp stem cells in chitosan/gelatin scaffolds for enhanced orofacial bone regeneration. Dent Mater. 2019;35:310–27. 30527589 10.1016/j.dental.2018.11.025

[35] Masutani EM, Kinoshita CK, Tanaka TT, Ellison AKD, Yoza BA. Increasing thermal stability of gelatin by UV-induced cross-linking with glucose. Int J Biomater. 2014;2014: 979636. 24963297 10.1155/2014/979636 PMC4055452

[36] Song Y, Nagai N, Saijo S, Kaji H, Nishizawa M, Abe T. In situ formation of injectable chitosan-gelatin hydrogels through double crosslinking for sustained intraocular drug delivery. Mater Sci Eng C. 2018;88:1–12. 10.1016/j.msec.2018.02.022 29636124

[37] Yao R, Zhang R, Luan J, Lin F. Alginate and alginate/gelatin microspheres for human adipose-derived stem cell encapsulation and differentiation. Biofabrication. 2012;4:1–11. 10.1088/1758-5082/4/2/025007 22556122

[38] Daly AC, Riley L, Segura T, Burdick JA. Hydrogel microparticles for biomedical applications. Nat Rev Mater. 2020;5:20–43. 34123409 10.1038/s41578-019-0148-6 PMC8191408

[39] Wei DX, Dao JW, Liu HW, Chen GQ. Suspended polyhydroxyalkanoate microspheres as 3D carriers for mammalian cell growth. Artif Cells, Nanomed Biotechnol. 2018;46:473–83. 29653500 10.1080/21691401.2018.1459635

[40] Samiei M, Abdollahinia ED, Amiryaghoubi N, Fathi M, Barar J, Omidi Y. Injectable thermosensitive chitosan/gelatin hydrogel for dental pulp stem cells proliferation and differentiation. BioImpacts. 2023;13:63–72. 36816999 10.34172/bi.2022.23904 PMC9923811

[41] Azhar FF, Olad A, Salehi R. Fabrication and characterization of chitosan-gelatin/nanohydroxyapatite- polyaniline composite with potential application in tissue engineering scaffolds. Des Monomers Polym. 2014;17:654–67.

[42] Phutane P, Telange D, Agrawal S, Gunde M, Kotkar K, Pethe A. Biofunctionalization and applications of polymeric nanofibers in tissue engineering and regenerative medicine. Polymers (Basel). 2023;15:1–38. 10.3390/polym15051202 PMC10007057 36904443

[43] Leite ML, Soares DG, Anovazzi G, Anselmi C, Hebling J, de Souza Costa CA. Fibronectin-loaded collagen/gelatin hydrogel is a potent signaling biomaterial for dental pulp regeneration. J Endod. 2021;47:1110–7. 33887309 10.1016/j.joen.2021.04.009

[44] Maji K, Dasgupta S, Pramanik K, Bissoyi A. Preparation and evaluation of gelatin-chitosan-nanobioglass 3D porous scaffold for bone tissue engineering. Int J Biomater. 2016;2016:9825659. 26884764 10.1155/2016/9825659 PMC4738941

[45] Obregon F, Vaquette C, Ivanovski S, Hutmacher DW, Bertassoni LE. Three-dimensional bioprinting for regenerative dentistry and craniofacial tissue engineering. J Dent Res. 2015;94:143S-152S. 26124216 10.1177/0022034515588885

[46] Gupte MJ, Ma PX. Nanofibrous scaffolds for dental and craniofacial applications. J Dent Res. 2012;91:227–34. 21828356 10.1177/0022034511417441 PMC3275331

[47] Xing Y, Qing X, Xia H, Hao S, Zhu H, He Y, et al. Injectable hydrogel based on modified gelatin and sodium alginate for soft-tissue adhesive. Front Chem. 2021;9:1–8. 10.3389/fchem.2021.744099 PMC8493121 34631665

[48] Chang W, Tai A, Tsai N, Li YE. An injectable hybrid gelatin methacryloyl (GelMA)/Phenyl isothiocyanate-modified gelatin (Gel-Phe) bioadhesive for oral/dental hemostasis applications. Polymers (Basel). 2021;13:2386. 34301143 10.3390/polym13142386 PMC8309571

[49] Ribeiro JS, Bordini EAF, Ferreira JA, Mei L, Dubey N, Fenno JC, et al. Injectable MMP-responsive nanotube-modified gelatin hydrogel for dental infection ablation. ACS Appl Mater Interfaces. 2020;12:16006–17. 32180395 10.1021/acsami.9b22964 PMC7370252

[50] Shahi S, Dehghani F, Abdolahinia ED, Sharifi S, Ahmadian E, Gajdács M, et al. Effect of gelatinous spongy scaffold containing nano-hydroxyapatite on the induction of odontogenic activity of dental pulp stem cells. J King Saud Univ—Sci. 2022;34: 102340.

[51] Golubevas R, Stankeviciute Z, Zarkov A, Golubevas R, Hansson L, Raudonis R, et al. Acrylate-gelatin-carbonated hydroxyapatite (cHAP) composites for dental bone-tissue applications. Mater Adv. 2020;1:1675–84.

[52] Theocharis AD, Skandalis SS, Gialeli C, Karamanos NK. Extracellular matrix structure. Adv Drug Deliv Rev. 2016;97:4–27. 26562801 10.1016/j.addr.2015.11.001

[53] Saotome T, Shimada N, Matsuno K, Nakamura K, Tabata Y. Gelatin hydrogel nonwoven fabrics of a cell culture scaffold to formulate 3-dimensional cell constructs. Regen Ther. 2021;18:418–29. 34722838 10.1016/j.reth.2021.09.008 PMC8524245

[54] Kumar VB, Tiwari OS, Finkelstein-Zuta G, Rencus-Lazar S, Gazit E. Design of functional RGD peptide-based biomaterials for tissue engineering. Pharmaceutics. 2023;15:1–17. 10.3390/pharmaceutics15020345 PMC9967156 36839667

[55] Wang F, Li Y, Shen Y, Wang A, Wang S, Xie T. The functions and applications of RGD in tumor therapy and tissue engineering. Int J Mol Sci. 2013;14:13447–62. 23807504 10.3390/ijms140713447 PMC3742196

[56] Garcia-Orue I, Santos-Vizcaino E, Etxabide A, Uranga J, Bayat A, Guerrero P, et al. Development of bioinspired gelatin and gelatin/chitosan bilayer hydrofilms for wound healing. Pharmaceutics. 2019;11:1–18. 10.3390/pharmaceutics11070314 PMC6680716 31277455

[57] Herliana H, Yusuf HY, Laviana A, Wandawa G, Cahyanto A. Characterization and analysis of chitosan-gelatin composite-based biomaterial effectivity as local hemostatic agent: a systematic review. Polymers (Basel). 2023;15:575. 36771876 10.3390/polym15030575 PMC9920696

[58] Li X, Zhang J, Kawazoe N, Chen G. Fabrication of highly crosslinked gelatin hydrogel and its influence on chondrocyte proliferation and phenotype. Polymers (Basel). 2017;9:309–22. 30970984 10.3390/polym9080309 PMC6418707

[59] Afewerki S, Sheikhi A, Kannan S, Ahadian S, Khademhosseini A. Gelatin-polysaccharide composite scaffolds for 3D cell culture and tissue engineering: Towards natural therapeutics. Bioeng Transl Med. 2019;4:96–115. 30680322 10.1002/btm2.10124 PMC6336672

[60] Covarrubias C, Cádiz M, Maureira M, Celhay I, Cuadra F, von Marttens A. Bionanocomposite scaffolds based on chitosan–gelatin and nanodimensional bioactive glass particles: in vitro properties and in vivo bone regeneration. J Biomater Appl. 2018;32:1155–63. 29451421 10.1177/0885328218759042

[61] Anthon SG, Valente KP. Vascularization strategies in 3D cell culture models: from scaffold-free models to 3D bioprinting. Int J Mol Sci. 2022;23:14582. 36498908 10.3390/ijms232314582 PMC9737506

[62] de Souza MF, da Silva HN, Rodrigues JFB, Macêdo MDM, de Sousa WJB, Barbosa RC, et al. Chitosan/gelatin scaffolds loaded with jatropha mollissima extract as potential skin tissue engineering materials. Polymers (Basel). 2023;15:603. 36771903 10.3390/polym15030603 PMC9921636

[63] Lan G, Lu B, Wang T, Wang L, Chen J, Yu K, et al. Chitosan/gelatin composite sponge is an absorbable surgical hemostatic agent. Colloids Surfaces B Biointerfaces. 2015;136:1026–34. 26590895 10.1016/j.colsurfb.2015.10.039

[64] Edmondson R, Broglie JJ, Adcock AF, Yang L. Three-dimensional cell culture systems and their applications in drug discovery and cell-based biosensors. Assay Drug Dev Technol. 2014;12:207–18. 24831787 10.1089/adt.2014.573 PMC4026212

[65] Åkerlund E, Gutyte G, Moussaud-Lamodière E, Lind O, Bwanika HC, Lehti K, et al. The drug efficacy testing in 3D cultures platform identifies effective drugs for ovarian cancer patients. npj Precis Oncol. 2023;7:111. 37907613 10.1038/s41698-023-00463-z PMC10618545

[66] Chakraborty J, Fernández-Pérez J, Van Kampen KA, Roy S, Ten Brink T, Mota C, et al. Development of a biomimetic arch-like 3D bioprinted construct for cartilage regeneration using gelatin methacryloyl and silk fibroin-gelatin bioinks. Biofabrication. 2023;15: 035009. 10.1088/1758-5090/acc68f 36947889

[67] Samadian H, Zamiri S, Ehterami A, Farzamfar S, Vaez A, Khastar H, et al. Electrospun cellulose acetate/gelatin nanofibrous wound dressing containing berberine for diabetic foot ulcer healing: in vitro and in vivo studies. Sci Rep. 2020;10:8312. 32433566 10.1038/s41598-020-65268-7 PMC7239895

[68] Rickel AP, Deng X, Engebretson D, Hong Z. Electrospun nanofiber scaffold for vascular tissue engineering. Mater Sci Eng C. 2021;129: 112373. 10.1016/j.msec.2021.112373 PMC8486306 34579892

[69] Roldán E, Reeves ND, Cooper G, Andrews K. Can we achieve biomimetic electrospun scaffolds with gelatin alone? Front Bioeng Biotechnol. 2023;11:1–17. 10.3389/fbioe.2023.1160760 PMC10368888 37502104

[70] Reddy MSB, Ponnamma D, Choudhary R, Sadasivuni KK. A comparative review of natural and synthetic biopolymer composite scaffolds. Polymers (Basel). 2021;13:1105. 33808492 10.3390/polym13071105 PMC8037451

[71] Campiglio CE, Negrini NC, Farè S, Draghi L. Cross-linking strategies for electrospun gelatin scaffolds. Materials (Basel). 2019;12:2476. 31382665 10.3390/ma12152476 PMC6695673

[72] Van Den Bulcke AI, Bogdanov B, De Rooze N, Schacht EH, Cornelissen M, Berghmans H. Structural and rheological properties of methacrylamide modified gelatin hydrogels. Biomacromol. 2000;1:31–8. 10.1021/bm990017d 11709840

[73] Contessi Negrini N, Angelova Volponi A, Sharpe PT, Celiz AD. Tunable cross-linking and adhesion of gelatin hydrogels via bioorthogonal click chemistry. ACS Biomater Sci Eng. 2021;7:4330–46. 34086456 10.1021/acsbiomaterials.1c00136

[74] Echave MC, Hernáez-Moya R, Iturriaga L, Pedraz JL, Lakshminarayanan R, Dolatshahi-Pirouz A, et al. Recent advances in gelatin-based therapeutics. Expert Opin Biol Ther. 2019;19:773–9. 31009588 10.1080/14712598.2019.1610383

[75] Lutzweiler G, Halili AN, Vrana NE. The overview of porous, bioactive scaffolds as instructive biomaterials for tissue regeneration and their clinical translation. Pharmaceutics. 2020;12:1–29. 10.3390/pharmaceutics12070602 PMC7407612 32610440

[76] Velasco MA, Narváez-Tovar CA, Garzón-Alvarado DA. Design, materials, and mechanobiology of biodegradable scaffolds for bone tissue engineering. Biomed Res Int. 2015;2015:1–21. 10.1155/2015/729076 PMC4391163 25883972

[77] Amiryaghoubi N, Samiei M, Fathi M, Barar J, Omidi Y, Dalir AE. Injectable thermosensitive chitosan/gelatin hydrogel for dental pulp stem cells proliferation and differentiation. Bioimpacts. 2022;13:63–72. 36816999 10.34172/bi.2022.23904 PMC9923811

[78] Ramezani M, Mohd RZ. 4D printing in biomedical engineering: advancements, challenges, and future directions. J Funct Biomater. 2023;14:347. 37504842 10.3390/jfb14070347 PMC10381284

[79] Liu S, Yu JM, Gan YC, Qiu XZ, Gao ZC, Wang H, et al. Biomimetic natural biomaterials for tissue engineering and regenerative medicine: new biosynthesis methods, recent advances, and emerging applications. Mil Med Res. 2023;10:1–30. 36978167 10.1186/s40779-023-00448-w PMC10047482

[80] Tabatabaei F, Moharamzadeh K, Tayebi L. Fibroblast encapsulation in gelatin methacryloyl (GelMA) versus collagen hydrogel as substrates for oral mucosa tissue engineering. J Oral Biol Craniofacial Res. 2020;10:573–7. 10.1016/j.jobcr.2020.08.015 PMC7479286 32939336

[81] de Londero C, Pagliarin CML, Felippe MCS, Felippe WT, Danesi CC, Barletta FB. Histologic analysis of the influence of a gelatin-based scaffold in the repair of immature dog teeth subjected to regenerative endodontic treatment. J Endod. 2015;41:1619–25. 26255965 10.1016/j.joen.2015.01.033

[82] Jones TD, Kefi A, Sun S, Cho M, Alapati SB. An optimized injectable hydrogel scaffold supports human dental pulp stem cell viability and spreading. Adv Med. 2016;2016:1–8. 10.1155/2016/7363579 PMC4884792 27294191

[83] Bhatnagar D, Bherwani AK, Simon M, Rafailovich MH. Biomineralization on enzymatically cross-linked gelatin hydrogels in the absence of dexamethasone. J Mater Chem B. 2015;3:5210–9. 32262596 10.1039/c5tb00482a

[84] Qu T, Jing J, Jiang Y, Taylor RJ, Feng JQ, Geiger B, et al. Magnesium-containing nanostructured hybrid scaffolds for enhanced dentin regeneration. Tissue Eng—Part A. 2014;20:2422–33. 24593189 10.1089/ten.tea.2013.0741 PMC4161063

[85] Chen G, Chen J, Yang B, Li L, Luo X, Zhang X, et al. Combination of aligned PLGA/Gelatin electrospun sheets, native dental pulp extracellular matrix and treated dentin matrix as substrates for tooth root regeneration. Biomaterials. 2015;52:56–70. 25818413 10.1016/j.biomaterials.2015.02.011

[86] Sharifi S, Samiei M, Dalir E, Khalilov R, Shahi S, Maleki DS. Gelatin-hydroxyapatite nano-fibers as promising scaffolds for guided tissue regeneration (GTR): preparation, assessment of the physicochemical properties and the effect on mesenchymal stem cells. J Adv Periodontol Implant Dent. 2020;12:25–30. 35919304 10.34172/japid.2020.001 PMC9327463

[87] Shahi S, Sharifi S, Khalilov R, Dizaj SM, Abdolahinia ED. Gelatin-hydroxyapatite fibrous nanocomposite for regenerative dentistry and bone tissue engineering. Open Dent J. 2022;16:1–10.

[88] Choi D, Qiu M, Hwang YC, Oh WM, Koh JT, Park C, et al. The Effects of 3-dimensional bioprinting calcium silicate cement/methacrylated gelatin scaffold on the proliferation and differentiation of human dental pulp stem cells. Materials (Basel). 2022;15:2170. 35329621 10.3390/ma15062170 PMC8948861

[89] Ducret M, Costantini A, Gobert S, Farges JC, Bekhouche M. Fibrin-based scaffolds for dental pulp regeneration: from biology to nanotherapeutics. Eur Cell Mater. 2021;41:1–14. 33387443 10.22203/eCM.v041a01

